# Radiologically Assessed Sex-Specific Left-Hand Digit Ratio (2D:4D) in Caucasian Children and Adolescents from South Germany

**DOI:** 10.1007/s10508-025-03254-8

**Published:** 2025-11-14

**Authors:** Katharina A. Schiergens, Ursula Berger, Ilja Dubinski, Birgit Kammer, Heinrich Schmidt

**Affiliations:** 1 Division of Pediatric Endocrinology and Diabetes, Department of Pediatrics, University Childrens Hospital, Ludwig-Maximilians-University Munich, Dr. von Hauner Childrens Hospital, Munich, Germany; 2 Institute for Medical Information Processing, Biometry, and Epidemiology, Ludwig-Maximilians-University Munich, Munich, Germany; 3 Department of Pediatric Radiology, Ludwig-Maximilians-University Munich, Dr. von Hauner Childrens Hospital, Munich, Germany

**Keywords:** Digit ratio, Finger ratios, Children, Adolescents, 2D:4D

## Abstract

The relative length of the 2nd (index) and 4th (ring) finger, i.e., digit ratio (or 2D:4D) is known to be different in males and females. Few radiologically assessed data on 2D:4D for children and adolescents are available. The aim of this study was to create a local database (MuC-2020) for the radiologically assessed sex-specific left-hand 2D:4D, and to compare this data to historical cohorts from three radiographic atlases of skeletal development of the hand and wrist. Therefore, left-hand X-ray images of 169 female and 199 male children and adolescents (age: 8–18 years) who presented at our endocrine unit in 2019 and 2020 mostly for reasons of predicting final height were analyzed. The 2D:4D ratio was calculated and compared with ratios determined from X-ray images of patients > 8 years of the atlases Greulich and Pyle (G&P, data from USA, 1936–1942), Gilsanz and Ratib (G&R, data from USA, 1980–2000), and Thiemann and Nitz (T&N, data from German Democratic Republic, 1977). Female individuals showed a higher 2D:4D ratio with a mean of 0.936 compared to males (0.919, *p* < 0.001). When comparing to historical data, the MuC-2020 2D:4D ratio significantly differed (females, G&P, *p* = 0.029; males, G&P *p* < 0.001, G&R, *p* = 0.018), with an overall increasing 2D:4D trend over time. The results of this analysis underpin the binarity of sex-specific left-hand 2D:4D in children and adolescents, and show differences to previously published cohorts suggesting an increasing 2D:4D ratio trend over the last decades. The underlying reasons are unknown, but environmental factors may be a reason (e.g., endocrine disrupting substances).

## Introduction

There is a long-standing tradition of paying attention to finger length in humans starting with the index digit. In the 19th century, Pfitzner (1893) was the first to publish data regarding the length of tubular bones of the hand. He could demonstrate that comparing the length of ring digit (4D) with middle digit (3D), and index digit (2D) with 3D, respectively, the index digit (2D) is shorter in the majority of males as compared to females, while the length of 4D is not influenced by the sex of the individual. Wood-Jones (1941) postulated that a relatively long D2 may solely be due to an increase in the relative length of it, irrespective of the length of the metacarpal and that index digit length varies independently of all other elements of the hand. Schultz (1926) found that sex differences in index finger length could be observed as soon as in the third month of life. Garn et al. (1972) stated that the ratio between phalanges of one digit ray stabilizes after the age of 4–9 years.

Regarding the ring digit (4D), it was suggested that fetal testosterone and estrogen influence the formation of the 2D:4D ratio. Low 2D:4D ratios indicate high fetal testosterone and low fetal estrogen exposure, while a high 2D:4D ratio indicates low fetal testosterone and high estrogen (Manning et al., 1998, 2002). The sex difference in this ratio is now better understood (Butovskaya et al., 2023; Galis et al., 2010; Malas et al., 2006; Manning & Fink, 2023; Trivers et al., 2020). Evidence suggests that the sexual dimorphism in 2D:4D driven by testosterone and estrogen concentrations refers to a narrow developmental window at the end of the first trimester (Trivers et al., 2020) although it may change in postnatal life as the fingers grow. This change appears to result in a gentle 2D:4D increase (McIntyre et al., 2005; Trivers et al., 2006). Radiographic studies have consistently corroborated sexual 2D:4D dimorphism with boys having lower 2D:4D than girls (e.g., in young Tuvans; Butovskaya et al., 2023). Furthermore, ethnic differences have been demonstrated (e.g., between White children and those of Afro-Caribbean descent; Trivers et al., 2020).

Zheng and Cohn (2011) could demonstrate that the amount of fetal testosterone and estrogen controls 2D:4D in mice, such as high testosterone increased the length of 4D (thereby leading to reduction of the ratio) and high estrogen reduced 4D growth–leading to an increased ratio. This marked effect of testosterone and estrogen on 4D can be explained by the fact that the mouse fetal digit is richly supplied with receptors for both hormones. Auger et al. (2013) exposed rat fetuses to normal levels of estrogen and anti-androgenic disruptors. In comparison to controls, male rats exposed to disruptors showed feminized digit ratios. The authors emphasized the potential role of 2D:4D as a biomarker of prenatal exposure to environmental levels of endocrine disruptors.

As Galis et al. (2010) and Malas et al., (2006) stated, sex differences in 2D:4D ratio are apparent by week 14 of fetal life. Still, this does not rule out an influence of peri- and postnatal testosterone levels. Knickmeyer et al. (2011) could demonstrate that prenatal as well as postnatal (in the first 2 years of life) exposure to testosterone does impact the 2D:4D ratio in males. In general, non-radiological measurement of finger length may be a useful tool to estimate the finger ratio but is more prone to bias by positioning than direct measurement on X-ray images.

Under all these aspects mentioned above the aim of our study was to determine the actual, sex-specific, radiological 2D:4D ratio for caucasian children and adolescents living in the area of Munich (South Germany) and to compare our data with data collected from three different radiological atlases for bone age assessment (Gilsanz, 2005; Greulich, 1959; Thiemann, 1991).

## Method

### Subjects

Patients were recruited from the endocrine and diabetes outpatient unit of the Dr von Hauner Children’s Hospital during the time period of 2019–2020 (about 2000 patients/year) –the MuC-2020 cohort. Patients having an X-ray imaging of the left hand for clinical purpose were selected. For each patient the complete electronic file was provided, and the last medical report containing all diagnoses was evaluated. The following group of patients was included in the study: Caucasians, patients aged 8 to 18 years, with diagnosis of Constitutional Delay of Puberty and Growth, suspected precocious puberty, late puberty, diabetes mellitus or suspected (but not confirmed) growth hormone deficiency. Predicting final height was the primary clinical objective and diagnostic aim for all individuals. We excluded children younger than 8 years, non-caucasians and patients with skeletal dysplasia, chromosomal anomalies, genetic defects, genetic forms of hematologic diseases, syndromatic disorders, congenital hypopituitarism, congenital adrenal hyperplasia, familial short stature, born small for gestational age, gender disphoria, visible malformations of bones in the present X-ray image and with estimated final height outside familial target height. A total of 169 females and 199 males were included in the analysis.

### Procedure

All radiographs of the left hand were acquired with a Philips Optimus Bucky Diagnost (Philips Medical Systems, Hamburg, Germany) with a focus size of 0.6 and a focus-detector distance of 100 cm. No grid or additional filtering was used. Radiographic settings were as follows: Tube voltage 60 kV; current-time product (mAs) 0.8. All images were obtained in dorso-volar projection by hand in direct contact with the image plate. Radiographs were digitally acquired using radiographic image plates and read using an Agfa DX-G processing unit (Agfa, Mortsel, Belgium). Measurements were performed on a scale of 1:0.75–1:1.25.

### Measures

The overall length of each digit (2D and 4D) including the epiphysis was measured as reported previously (from the proximal tip of the proximal phalanx to the distal tip of the distal phalanx) using a ruler (KUM Germany) accurate to 0.5 mm (Manning et al., 2000). Digit lengths were measured twice by one rater with a maximum measurement error of ±1 mm. The mean of the two measurements was taken as the final result. 2D and 4D were measured in the atlas of Greulich & Pyle, Gilsanz & Ratib, and Thiemann & Nitz in the published original size (Gilsanz, 2005; Greulich, 1959; Thiemann, 1991). Only X-ray images of children elder than 8 years were considered. The ratio 2D:4D was calculated.

### Statistical Analysis

The distribution of 2D:4D ratios of the MuC-2020 cohort as well as three external data sources were described separately for males and females reporting lower and upper quartiles (q1, median, q3) as well as means with standard deviations (SD). The results were displayed in boxplots. To quantify the differences in mean 2D:4D ratios between MuC-2020 and the three data sources, linear regression was applied. Differences between mean 2D:4D ratios were tested based on two-sided t-tests and considered significant at a α-level of < 0.05. All analyses were performed with the statistical package R version 3.6.2 (R Team, 2019).

## Results

**Fig. 1:**
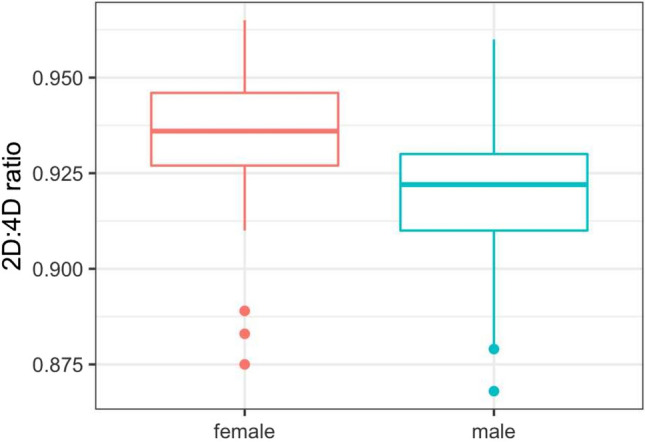
Comparison of 2D:4D ratio of females and males of the Munich Cohort (MuC-2020)

X-ray data were available for the MuC-2020 cohort for 169 female participants and for 199 male participants. Females showed a mean 2D:4D ratio of 0.936. The 2D:4D digit ratio of male participants (0.919) was found to be significantly lower (*p* < 0.001) (Table 1 and Fig. 1). The 2D:4D ratios of the four different cohorts show an increase over time.

**Table 1:** 2D:4D ratios of female and male participants of the Munich Cohort (MuC-2020)

Sex	n	q1	Median	q3	Mean	SD	*p*-value^a^
MuC-2020 female	169	0.927	0.936	0.946	0.936	0.014	
MuC-2020 male	199	0.910	0.922	0.930	0.919	0.015	**< 0.001**

^a^*p*-value of the *t*-test comparing females to males; significant values with *p* < 0.05 in bold

**Fig. 2:**
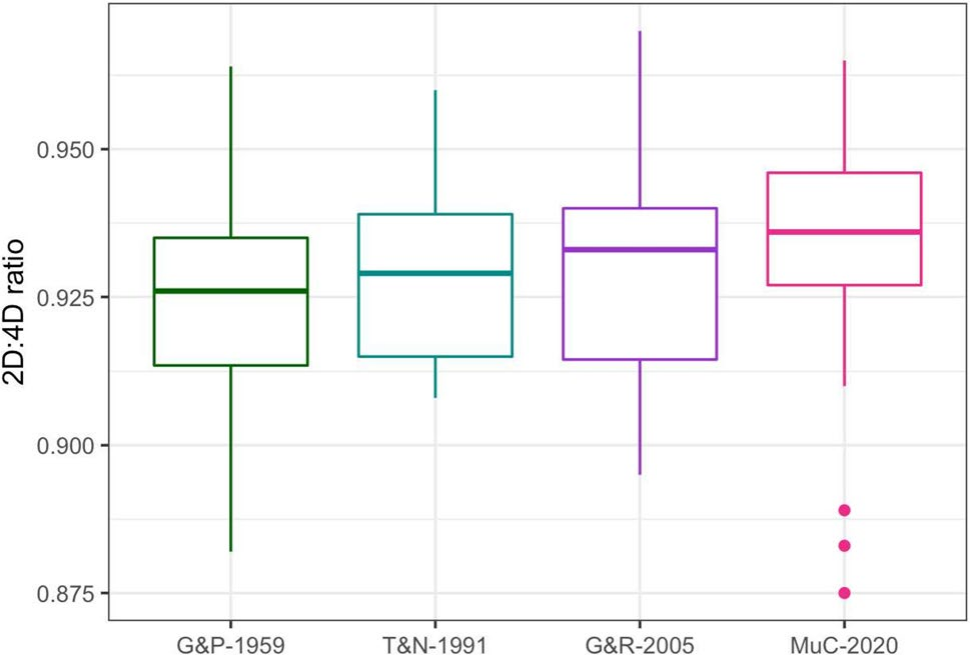
2D:4D ratios of females from different cohorts (G&P-1959: Greulich & Pyle; T&N-1991: Thiemann & Nitz; G&R-2005: Gilsanz & Ratib; MuC-2020: Munich Cohort 2020).

**Fig. 3:**
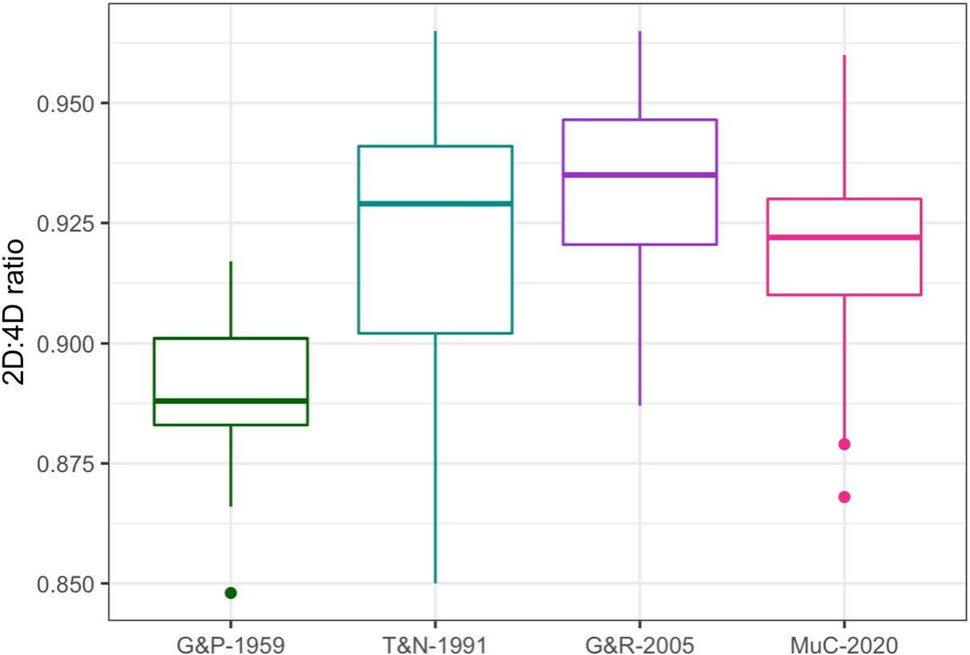
2D:4D ratios of males from different cohorts (G&P-1959: Greulich & Pyle; T&N-1991: Thiemann & Nitz; G&R-2005: Gilsanz & Ratib; MuC-2020: Munich Cohort 2020).

Females measured by Greulich and Pyle (1959) showed a significantly lower 2D:4D ratio of 0.927 with a mean difference to the Munich cohort of −0.009 (*p* = 0.030) (Table 2 and Fig. 2). Males measured from the atlas by Greulich and Pyle (1959) showed an even more distinct difference in the 2D:4D digit ratio compared to MuC-2020: with a mean difference of −0.029 their 2D:4D digit ratios were markedly lower than that of MuC-2020. In contrast, the measures derived from Gilsanz and Ratib (2005) were higher by 0.013 (Table 3 and Fig. 3). No consistent trend in sex differences over time was observed across the four groups.

**Table 2:** 2D:4D ratios of females from different cohorts (G&P-1959: Greulich & Pyle; T&N-1991: Thiemann & Nitz; G&R-2005: Gilsanz & Ratib; MuC-2020: Munich Cohort 2020)

Study group	n	q1	Median	q3	Mean	SD	*p* value^a^
MuC-2020	169	0.927	0.936	0.946	0.936	0.014	
G&P-1959	14	0.914	0.926	0.935	0.927	0.023	**0.029**
G&R-2005	11	0.915	0.933	0.940	0.930	0.022	0.188
T&N-1991	17	0.915	0.929	0.939	0.930	0.015	0.107

^a^*p* value of the *t*-test comparing differences to female participants of MuC-2020; significant values with *p* < 0.05 in bold

**Table 3:** 2D:4D ratios of males from different cohorts (G&P-1959: Greulich & Pyle; T&N-1991: Thiemann & Nitz; G&R-2005: Gilsanz & Ratib; MuC-2020: Munich Cohort 2020)

Study group	n	q1	Median	q3	Mean	SD	*p* value^a^
MuC-2020	199	0.910	0.922	0.930	0.919	0.015	
G&P-1959	15	0.883	0.888	0.901	0.890	0.019	**< 0.001**
G&R-2005	11	0.921	0.935	0.947	0.932	0.023	**0.018**
T&N-1991	17	0.902	0.929	0.941	0.924	0.028	0.260

^a^*p* value of the *t*-test comparing differences to male participants of MuC-2020; significant values with *p* < 0.05 in bold

## Discussion

There are only few research data on X-ray based measured 2D:4D ratios (Buck et al., 2003; Butovskaya et al., 2023; Peeters et al., 2013; Robertson et al., 2008; Trivers et al., 2020; Xi et al., 2014). This is, to our knowledge, the first study on radiologically determined 2D:4D ratio in a larger group of German children and adolescents as well as the first study comparing those ratios to those of historical cohorts of different decades of the last century thereby analyzing time trends.

The 2D:4D ratio represents an individual difference putatively related to prenatal and short postnatal gonadal hormonal exposure. A lower index digit is indicative of relatively higher testosterone than estrogen influence. Therefore 2D:4D ratios in both children and adolescents show substantial within-sex variability and a high degree of overlap between males and females. Of course, a part of the inter-individual variability is unrelated to sex steroid exposure. Despite the sex steroid and the non-sex steroid variability, radiologically assessed finger length and the 2D:4D ratio is a valuable asset to highlight the sexual dimorphism.

Peeters et al. (2013) found a 2D:4D ratio of 0.925 (SD, 0.019) in adolescent girls from a Flemish population. Of note, the measurements published in this study were carried out in 1979–1980. Vujovic et al. (2014) published data with a female left-hand index of 0.945, and a male left-hand index of 0.935 in control persons. Another study including Caucasian boys with autism found a left-hand index of 0.92 (Bloom et al., 2010). Vehmas et al. (2006) reported a female index of 0.925. It has been suggested that the ratio in African and Asian individuals is lower than in Caucasians, e.g., a female digit ratio of 0.93 and a male digit ratio of 0.92 (Manning et al., 2007; Xi et al., 2014). A recent study has demonstrated that compared to White children Afro-Caribbean childrens’ mean radiographic 2D:4D was lower also corroborating the well-known sexual dimorphism of 2D:4D ratio (Trivers et al., 2020). While it is widely accepted international standard to use an X-ray of the left hand and wrist in children to predict adult height and assess bone age, data published by Williams et al. (2000) saw a sex difference only on the right hand, but others have reported sex differences in digit ratios on both hands. Of note, these data were obtained from photocopies of subjects’ hands and not measured by X-ray.

When comparing our data with previous publications, one might take into consideration that Greulich and Pyle included X-ray images of TW Todd’s (1937) atlas, augmented with X-rays in the consecutive years. The subjects (more than 1000 children) were upcomings of the upper socio-economic class of Cleveland society and of European descent (Greulich, 1959). Thiemann and Nitz (1991) selected standards from 5200 X-rays of healthy children from the German Democratic Republic in 1977. Gilsanz and Ratib evaluated 522 (50% females) left-hand and wrist radiographs of healthy children and adolescents of European descendent (parents and both sets of grandparents). The radiographs were collected between 1980 and 2000 (Gilsanz, 2005). Thus, the populations being compared are different.

It is interesting to see that for both, females and males, the MuC-2020 cohort data of 2D:4D ratios are markedly higher when comparing to those of Greulich and Pyle (1959) (female: 0.936 vs. 0.927; male: 0.919 vs. 0.890). For boys, the ratios derived from the atlases of Gilsanz and Ratib as well as Thiemann and Nitz were as well significantly higher than those of Greulich and Pyle 1959 (0.932 and 0.924 vs. 0.890). Zheng and Cohn (2011) were the first who demonstrated that an antiandrogen in males or an antiestrogen in females displaces the 2D:4D ratios towards a feminized ratio in males and a masculinized in females.

Hence, the current literature and our data suggest that the 2D:4D ratio is variable depending on the population and has changed (overall increased) during the last 80 years. The underlying drivers for this phenomenon are unclear, but likely biological, environmental and social factors may have played a role suggesting a complex interplay of prenatal hormone exposure potentially influenced by environmental factors such as toxins, health conditions including nutrition, maternal health, societal changes, and further exposures (Manning et al., 2022a, b; Sitek et al., 2022; Wainstock et al., 2016). As Auger et al. (2013) could demonstrate in rats, the developing digits are a key target of estrogen-like and antiandrogenic chemicals (genistein, bisphenol A and vinclozolin) at low environmental doses. They also found that the digit ratios were feminized in the second generation sired by fathers exposed to bisphenol A. When mothers were exposed to a toxicant during pregnancy the developing embryo (F1) and the developing germline that will give rise to the following generation (F2) were also directly exposed. Their findings in F2 thus indicate that BPA or other endocrine active substances (EASs) may modify the epigenome of the F2 generation. McMechan et al. (2004) demonstrated that alcohol, as an antiandrogen, is acting as an endocrine disrupter thereby influencing digit ratios as well.

Limitations of our study include bias by using radiographs of a patient cohort which might not entirely reflect the general population, despite the fact that we were very restrictive with regard to inclusion and exclusion criteria. Still, this may not reflect an entirely “healthy” cohort although the diagnosis CDGP is considered a normal variant of development rather than a pathological condition, i.e., a variation in the timing of growth and pubertal onset. Another limitation represents the low number of data points derived from individuals of the three atlases with respective impact on comparative analysis. On the other hand, it is possible that the X-ray pictures selected and published in the atlases underlie a selection bias as well since their authors had chosen representative cases regarding growth plates for each of the age and gender groups.

The strength of our study is that we used standardized radiographs, we calculated the 2D:4D ratio using left-hand radiographs performed by only one radiological (inhouse) institute and only one rater measured and calculated the digit ratio ensuring consistency. It would be interesting to measure the original X-ray images of great longitudinal studies like London Longitudinal Series, Harpenden Growth Study and the Zurich Longitudinal Study, and to compare these more recently performed studies with our data.

## Conclusion

Radiologically assessed 2D:4D ratio in children older than eight years is sex-specific. This digit ratio seems to be population-dependent. Our data and the literature suggest that during the last eight decades this ratio appears to have increased. Multiple factors including exposures such as maternal health, nutritional factors, toxins and endocrine disrupting substances may be discussed as underlying drivers.

## Data Availability

The study data and statistical analyses are available on request.

## Code Availability

R code and statistical scripts are available on request.
